# Developing and using a full-population resource from Washington State administrative data

**DOI:** 10.23889/ijpds.v9i5.2904

**Published:** 2024-09-18

**Authors:** Jennifer L Romich, Shannon Harper, Sofia G Ayala, Callie Freitag, Elizabeth Pelletier, Geraldine Germain, Tagoipah Mathno

**Affiliations:** 1 University of Washington

In the U.S. context, state-level integrated data systems are often built around system domains, such as P20 data systems which integrate student education data from preschool through higher education. This paper presents an innovative effort to develop and deploy an integrated data system capturing administrative records for the full (adult) population of a state. This effort—WashPop—is a new statewide data repository for research and policy analysis in Washington State that includes a longitudinal core data file, combining microdata from state employment, voting, licensing, public assistance, and vital statistics systems, to which other data resources may be linked to answer timely, policy-relevant questions. In this paper, we describe the design of WashPop and expound on its practical utility to address state- and local-level well-being and equity challenges—including its ability to bridge informational gaps from survey data by speaking to the experiences of smaller populations like rural counties, smaller towns and cities, and small racial and ethnic groups. We present evidence showing how such linked data can replicate Census counts while providing fine-grained longitudinal earnings and residential histories. Our research further offers insights on the power of this data resource to augment incomplete record information on racial and ethnic identities for a sharper study of how these characteristics intersect with inequality. Case studies also illustrate the value of these data for informing the design of the state Paid Family and Medical Leave Act, evaluating the Seattle $15 Minimum Wage ordinance, and informing policy decisions around workforce and equity issues.

